# Drug-Tolerant Persister Cells and Tumor Dormancy in NSCLC: A New Frontier in Overcoming Therapeutic Resistance

**DOI:** 10.3390/cancers18050779

**Published:** 2026-02-28

**Authors:** Mumtu Lalla, Akshay Ratnani, Jihua Yang, Meng Wang, Haiying Cheng

**Affiliations:** 1 Department of Oncology, Montefiore Einstein Comprehensive Cancer Center, Albert Einstein College of Medicine, Montefiore Medical Center, 1695 Eastchester Road, Bronx, NY 10461, USA; 2 Internal Medicine, Jefferson Einstein Philadelphia Hospital, Philadelphia, PA 19141, USA

**Keywords:** drug-tolerant persister cells, therapy resistance, tumor dormancy, minimal residual disease, circulating tumor DNA, non–small cell lung cancer, tumor microenvironment

## Abstract

Targeted therapies and chemoimmunotherapy have transformed outcomes for non–small cell lung cancer (NSCLC), yet relapse remains common. Resistance is increasingly recognized to include an early, largely reversible phase in which a minor subpopulation survives lethal therapy through non-genetic adaptation. These drug-tolerant persister (DTP) cells may be quiescent or cycling, and provide a reservoir from which stable, genetically resistant clones can later emerge. In parallel, late recurrence may reflect tumor dormancy, in which disseminated or residual cells persist for prolonged periods under microenvironmental constraint and/or immune surveillance. This review integrates DTP and dormancy frameworks in NSCLC, summarizes mechanisms that sustain persistence (chromatin and transcriptional plasticity, stress signaling, metabolic rewiring, and stromal/immune protection), and highlights experimental models and translational readouts, including circulating tumor DNA (ctDNA)–based minimal residual disease (MRD) monitoring. We also discuss potential therapeutic concepts to prevent DTP formation, exploit persister liabilities, or enforce dormancy in minimal-disease settings. A mechanistically grounded understanding of these survival programs is essential for rational combinations and biomarker-guided trials aimed at durable remission.

## Simple Summary

Many lung cancers recur after an initial response to targeted therapy or chemo/immunotherapy. Increasing evidence suggests that relapse can arise from a small fraction of tumor cells that survive therapy without immediately acquiring classic resistance mutations. These drug-tolerant persister cells can be quiescent or slowly cycling and can later seed genetically resistant outgrowth. Tumor dormancy offers a complementary framework for long disease-free intervals followed by late recurrence, when disseminated or residual cells persist in specialized microenvironments and later reactivate. This review integrates persister and dormancy frameworks in NSCLC, highlights mechanisms that support persistence (epigenetic and transcriptional plasticity, stress responses, metabolic rewiring, and microenvironmental protection), and discusses experimental platforms and emerging clinical approaches to monitor residual disease, including ctDNA-based MRD. We outline potential therapeutic strategies intended to reduce or eliminate residual reservoirs and to improve the durability of remission.

## 1. Introduction

Lung cancer remains a leading cause of cancer-related mortality worldwide, and relapse after apparently effective therapy is common. The discovery of actionable genomic alterations and the development of targeted therapies and chemoimmunotherapy combinations have transformed the management of advanced NSCLC [1]. Nevertheless, durable remissions remain uncommon, particularly in oncogene-driven disease treated with tyrosine kinase inhibitors (TKIs), where residual disease frequently persists and ultimately progresses.

Relapse has traditionally been attributed to the selection of rare, pre-existing genetic resistance variants. However, multiple studies support an earlier, largely reversible phase in which a minor subpopulation survives lethal stress through non-genetic adaptation. Seminal work described a drug-tolerant state that emerges rapidly after therapy, persists across diverse agents, and can revert to drug sensitivity after drug withdrawal, in part through altered chromatin regulation and IGF1R signaling [2]. Subsequent work in lung cancer and other tumors linked early persistence to later acquisition of stable resistance and to clinically relevant minimal residual disease (MRD) [3,4,5,6].

In parallel, tumor dormancy has long been used to explain long disease-free intervals followed by late recurrence. Dormancy can present as quiescent single-cell arrest, angiogenic dormancy, or immune-mediated equilibrium [7]. Single-cell and lineage-tracing studies suggest partial overlap between persister and dormancy biology—both emphasize stress-adapted survival, microenvironmental dependence, and the potential for reactivation—yet the anatomical context and timescale often differ [7,8].

## 2. Features of Drug-Persister Cells, Tumor Dormancy, and Minimal Residual Disease

### 2.1. Drug-Tolerant Persister Cells

The term “persister” originated in microbiology, where a small fraction of bacteria survives lethal antibiotic exposure through a transient phenotypic state rather than heritable resistance [9,10,11]. In cancer, DTP cells are best treated as a functional designation: a rare subpopulation that remains viable during continuous therapy that eliminates the bulk tumor population, without requiring fixed resistance mutations at the outset, and that can regain drug sensitivity after drug withdrawal [2,5,12].

DTPs are heterogeneous. Many show reduced proliferation and stress-adaptation programs, but both non-cycling and cycling persister populations have been described [13]. Cells can undergo various biological adaptations to survive selective therapeutic pressures, such as embodying a stem-like phenotype, activating bypass signaling pathways (e.g., PI3K/AKT, IGFR1, AXL), and undergoing epithelial-mesenchymal transition (EMT) [12]. In *EGFR*-mutant lung cancer models, *EGFR* blockade can enrich ALDH-positive stem-like subpopulations via Notch3-dependent signaling [14], and AXL-associated partial EMT programs are repeatedly implicated in tolerance and resistance, including under osimertinib exposure [15]. Together, these data argue against a single persister marker set and support a state-based definition.

Two non–mutually exclusive models explain DTP emergence: selection of pre-existing “bet-hedging” lineages versus therapy-induced reprogramming. Empirical data support contributions from both, and persisters provide a reservoir from which genetically fixed resistance can ultimately arise [2,3,5].

### 2.2. Tumor Dormancy

Tumor dormancy refers to a clinically occult disease that persists for prolonged periods before overt recurrence. Dormancy can occur as cellular dormancy (single-cell quiescence) or as tumor-mass dormancy, imposed by extrinsic constraints such as limited angiogenesis or immune-mediated equilibrium [16,17]. Dormancy programs reflect both intrinsic signaling and microenvironmental cues, and they can coexist with immune evasion rather than durable immune control [17].

Persister and dormancy frameworks overlap conceptually, but they differ in context and timescale. DTP cells emerge under therapeutic pressure through non-genetic adaptations that promote a slow cycling or quiescent survival state, which can be reversible with drug withdrawal [2,5]. Dormant cells do not require prior therapy exposure; they can arise spontaneously and persist in a quiescent state within metastatic niches [16]. Accordingly, DTP states are most often discussed as early on-therapy survival states, whereas dormancy more often explains long latency between apparent disease control and later relapse [8,16].

### 2.3. Minimal Residual Disease as a Translational Bridge

MRD provides a clinically actionable setting where persister and dormancy biology converge. In localized NSCLC treated with curative intent, ctDNA-based strategies can detect MRD and, in some cohorts, anticipate relapse earlier than radiologic surveillance [18,19]. ctDNA kinetics, however, do not specify whether residual cells are dormant, slowly cycling, or expanding. A key translational need is to integrate MRD assays with state biomarkers from tumor tissue or blood to enable mechanism-based intervention trials [20].

## 3. Origins of Persister and Dormant States

**Figure 1:**
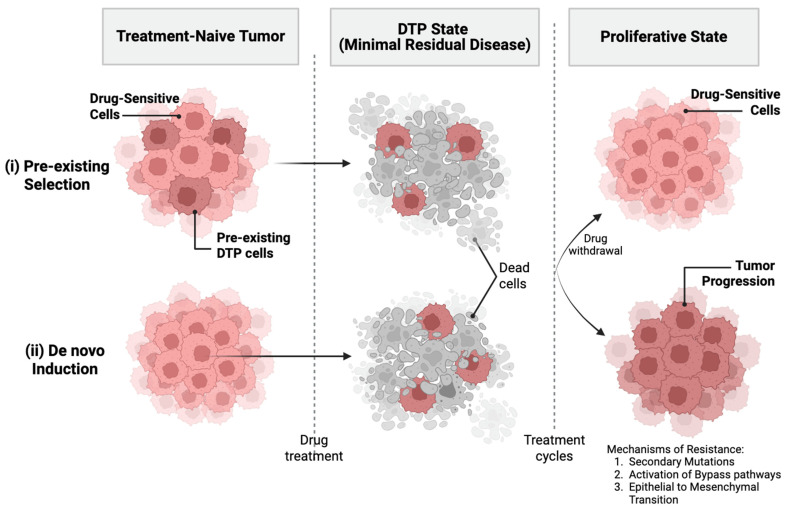
Generation of DTP cells. Schematic contrasting (i) selection of pre-existing “bet-hedging” lineages and (ii) therapy-induced state transitions. Following drug exposure, most drug-sensitive cells are eliminated, whereas DTPs survive in a transient, quiescent or slow-cycling state that constitutes minimal residual disease. Upon drug withdrawal, DTPs can re-enter proliferation with regained drug-sensitivity (top arrow). Alternatively, DTPs may serve as a reservoir for the acquisition of stable resistance, driving tumor progression (bottom arrow). Potential resistance mechanisms include secondary mutations, activation of bypass signaling pathways, and epithelial–mesenchymal transition. “Created in BioRender. Lalla, M. (2026). https://BioRender.com/tgbrwck”.

Lineage tracing and single-cell profiling support both pre-existing and therapy-induced origins of persister states. Cycling persisters can arise from lineages present before therapy, while non-cycling persisters can be enriched through therapy-induced reprogramming, where therapeutic pressures induce a biologic transition of a small subset of residual cells, driving their entry into the DTP state (Figure 1) [13,21]. Across cancers, MRD may progress through different routes toward relapse, including genetic fixation of resistance or prolonged non-genetic adaptation [5].

In NSCLC, DTP formation after *EGFR* inhibition is frequently accompanied by lineage plasticity and bypass signaling. ALDH activity can be required to maintain a drug-tolerant fraction [22]. AXL upregulation and EMT-like programs are repeatedly implicated in tolerant and resistant NSCLC states, including under osimertinib exposure [15]. In *ALK*-rearranged disease, YAP1-mediated survival programs have also been described under alectinib pressure [23], suggesting convergence on stress-adapted survival programs across oncogenic drivers.

In disseminated disease, dormancy programs can be induced or maintained by niche-derived signals. NR2F1 integrates epigenetic programs of quiescence and survival in disseminated tumor cells, including SOX9- and RARβ-linked quiescence programs [7]. Epigenetic priming (e.g., 5-azacytidine) can stabilize retinoid-driven dormancy programs through restored TGF-β–SMAD signaling and suppress metastatic outgrowth in preclinical models [24].

## 4. Mechanisms Supporting Drug Tolerance, Persistence, and Dormancy

**Figure 2:**
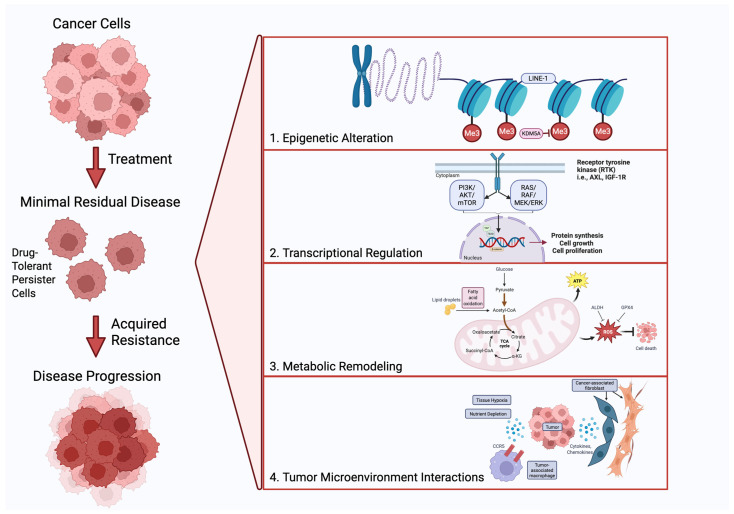
Mechanisms supporting DTP persistence and dormancy. Modular schematic: chromatin remodeling (KDM5A; LINE-1 repression), transcriptional bypass/lineage plasticity (AXL; IGF-1R; YAP/TEAD; Notch/WNT), metabolic rewiring (Oxidative Phosphorylation; GPX4/ferroptosis; Fatty Acid Oxidation), and microenvironmental/immune protection (CAF HGF/IGF; macrophage recruitment; hypoxia; NK-cell surveillance). “Created in BioRender. Lalla, M. (2026) https://BioRender.com/cns0ips”.

DTP phenotypes are supported by reversible survival programs that include epigenetic remodeling, transcriptional plasticity with bypass signaling, metabolic rewiring toward stress tolerance, and protection from stromal and immune compartments (Figure 2) [2,5,12]. These adaptations can be reversible, consistent with partial restoration of drug sensitivity after withdrawal in some models [2].

### 4.1. Epigenetic Remodeling and Chromatin State

Epigenetic plasticity is a core feature of early drug tolerance. Epigenetic alterations—including histone modifications, DNA methylation, and chromatin remodeling—modulate gene function without causing an inherent change in the DNA sequence, enabling cells with a flexibility to respond and adapt to environmental changes [1]. The canonical description of DTPs identified dependence on chromatin regulators and the H3K4 demethylase KDM5A/RBP2 [2]. Pharmacologic KDM5 inhibition reduces survival of drug-tolerant populations [25], and small-molecule KDM5A inhibition (including ryuvidine) limits gefitinib-tolerant states in *EGFR*-mutant models [26].

Other chromatin mechanisms can stabilize stress-adapted survival. Repression of stress-induced LINE-1 expression protects cancer cell subpopulations from lethal drug exposure, highlighting heterochromatin-associated buffering as a tolerance mechanism [27]. Overall, chromatin remodeling appears to enable rapid, reversible state switching under drug pressure.

### 4.2. Transcriptional Plasticity, Lineage Programs, and Stress Responses

Persisters often exhibit partial EMT-like programs, diminished lineage fidelity, and activation of bypass receptor tyrosine kinases. AXL upregulation is associated with tolerance and resistance in NSCLC, including under osimertinib exposure [15]. Insulin-like growth factor 1 receptor (IGF-1R) signaling can also support survival under *EGFR* pathway inhibition; acquired resistance mediated by loss of IGF-binding proteins provides an early example [28], and transient IGF-1R inhibition combined with osimertinib produced deep responses in preclinical models [29].

Developmental and stress-response pathways can reinforce “dormancy-like” survival. YAP/TEAD signaling promotes survival during combined *EGFR*/MEK inhibition and is associated with a therapy-induced dormant/senescent-like phenotype [30]. Stress circuitry can also accelerate adaptation: AURKA activation promotes evolution of resistance to third-generation *EGFR* inhibitors [31], supporting the idea that stress programs can enlarge the persister reservoir and facilitate eventual outgrowth.

WNT/β-catenin signaling regulates persister cell survival in a highly context-dependent manner. *EGFR* inhibition can induce Notch3-mediated activation of β-catenin, enabling the survival of DTP cells and early drug tolerance [32]. Separately, autocrine suppression of WNT signaling through DKK1 expression drives a quiescent, dormancy-like state, facilitating immune evasion and extended latency [8].

### 4.3. Metabolic Rewiring and Redox Homeostasis

Therapeutic stress can select for metabolic states that prioritize survival over growth. In *EGFR*-driven lung adenocarcinoma models, osimertinib exposure induces dependence on mitochondrial oxidative phosphorylation (OxPhos) rather than maintaining anaerobic glycolysis, which is favored by proliferating cancer cells [33]. OxPhos inhibition delays resistance in preclinical systems [33]. Persister survival is also shaped by redox control: persister-like states across cancers can acquire GPX4 dependence, creating sensitivity to ferroptosis upon GPX4 inhibition [34].

Lipid metabolism can contribute to tolerance in some contexts. Fatty-acid oxidation-dependent adaptation has been described during MAPK inhibitor stress in melanoma [35]. In lung adenocarcinoma, early cisplatin-tolerant transcriptional programs support the rapid establishment of stress-response and metabolic rewiring [36]. A practical implication is that metabolic liabilities are plausible but likely context- and therapy-specific, and should be validated across genotypes and in vivo microenvironments.

### 4.4. Tumor Microenvironmental Cues and Niche Protection

Persistence is not purely cell autonomous. Stromal interactions can buffer tumor cells from therapy by providing growth factors, survival cues, and protective niches. In *EGFR*-mutant lung cancer models, HGF-producing stromal fibroblasts induce resistance to *EGFR* TKIs [37]. Cancer-associated fibroblasts can promote EMT and *EGFR*-TKI resistance through HGF/IGF-linked pathways [38] and contribute to drug resistance through paracrine signaling [39].

Inflammatory niches may also stabilize residual disease; macrophage recruitment can promote recurrence in residual tumor settings [40]. In disseminated disease, dormancy can be maintained by niche constraints and immune surveillance. A Massagué-led study linked metastatic latency to immune evasion through autocrine inhibition of WNT signaling, connecting a quiescent-like state to reduced NK-cell clearance [8].

## 5. Persister Biology Across Oncogenic Drivers and Therapy Classes

Metabolic and survival adaptations that promote the emergence and persistence of DTPs have been observed across patients treated with targeted treatment for *ALK*-, *KRAS*-, and *ROS1*-mutated NSCLC. AXL overexpression with epithelial-mesenchymal transition has been seen in alectinib-resistant ALK-rearranged NSCLC [41]. *KRAS* G12C inhibition can alter redox homeostasis by increasing sensitivity to ferroptosis via GPX4 inhibition [42]. More broadly, repression of apoptosis through FAK-YAP signaling has been identified as a shared, cross-driver residual disease program during EGFR- ALK-, and KRAS- targeted therapies [43].

The kinetics of these adaptations vary by oncogenic driver and therapeutic context. Clinically, this variability is reflected in median progression-free survival (PFS), which can serve as a surrogate for time to resistance. For instance, osiertinib in the FLAURA trial achieved a median PFS of 18.9 months in first-line *EGFR*-mutated NSCLC [44], whereas adagrasib demonstrated a median PFS of 6.5 months in *KRAS*-mutated NSCLC in the KRYSTAL-1 trial [45]. Next-generation inhibitors such as repotrectinib, a ROS1 inhibitor, have shown greater durability, likely due to their ability to overcome additional resistance mutations [46].

Persister cells can also arise in response to immunotherapy. Immune persister cells (IPCs) exhibit epithelial-mesenchymal features characteristic of a stem-like state, and evade immune destruction through upregulation of PD-L1 and downregulation of tumor antigens [47,48]. Notably, both DTPs and IPCs develop decreased sensitivity to mitochondrial apoptosis, enabling survival under therapeutic stress and conferring cross-resistance to both targeted therapy and immunotherapy [48,49].

## 6. Experimental Models and Translational Readouts

Evaluating DTP cells presents fundamental challenges due to their transient nature, limited clinical accessibility, and remarkable plasticity, which enables transitions between different cellular states and results in substantial intratumoral phenotypic heterogeneity [5,49]. These features complicate the detection, characterization, and therapeutic targeting of DTP cells, limiting the clinical translation of strategies aimed at exploiting the DTP state.

### 6.1. Translational Challenges in DTP Identification

Mechanistic inference depends on operational definitions and model choice. Common approaches include prolonged drug exposure in cell lines, patient-derived organoids and xenografts, lineage barcoding, single-cell transcriptomics/epigenomics, and functional regrowth assays after drug withdrawal [5,12]. Single-cell profiling, lineage tracing, and CRISPR activation have been monumental in tracking the cellular trajectory of DTP formation during longitudinal treatment [5]. Lineage tracing additionally helps distinguish selection of pre-existing tolerant lineages from therapy-induced state transitions [13,21].

Past evaluation of DTPs has been limited to in vitro systems that preclude the ability to capture interactions between DTPs and the microenvironment [12,50]. In vivo models, such as organoids or genetically engineered mouse models, are essential to capture pharmacokinetic realities, immune pressure, and niche constraints relevant to persistence and dormancy [8]. Because no single model fully encompasses the complexity of DTP biology, integrating these systems with emerging technologies is critical. Recent advances in single-cell and spatial multiomic technologies and proteomics allow for recognition of metabolic adaptations and tumor microenvironment niches that influence DTP behavior [5,49]. Emerging tools—including AI-driven and machine learning analyses, live-cell imaging, and CRISPR screens—hold promise to further our understanding of DTP biology and uncover therapeutic vulnerabilities [5]. Furthermore, analysis of clinical trials of neoadjuvant targeted therapy, such as osimertinib (NCT04816838), provides a unique opportunity to examine DTP biology directly in resected patient tumors, bridging preclinical findings with clinical relevance [51].

### 6.2. Lack of Specific Biomarkers

The heterogeneous and plastic nature of DTP cells across different cancer types limits the generalizability of biomarkers and their reliable detection. Aldehyde dehydrogenase (ALDH) activity has previously been associated with drug-resistant disease recurrence; however, its tissue- and cancer-specific expression limits its generalizability, and the dynamic nature of DTP cells further limits its ability to accurately capture the full DTP population and predict clinical outcomes [52]. Alternative stem cell markers under evaluation include ABCB5, CD133, and CD271 [52]. Biomarkers (e.g., KDM5 and basal keratins) that identify DTP cells using their epigenetic and transcriptional signatures are also under investigation [53]. The context-dependent, plastic nature of the DTP state may ultimately necessitate a combination of biomarkers for comprehensive identification of these cells across different cancer types and therapeutic settings [5].

### 6.3. MRD as a Translational Bridge

**Figure 3:**
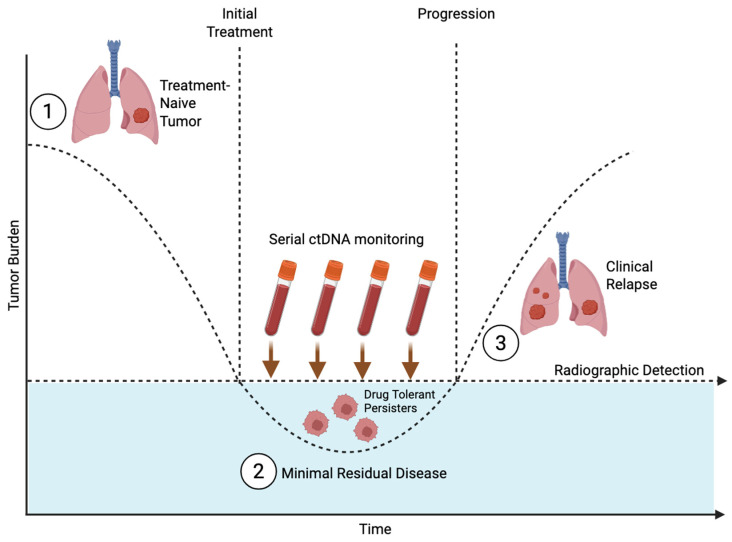
Minimal residual disease (MRD), a measurable bridge between early persistence and later relapse. Serial ctDNA monitoring has the potential to detect post-treatment MRD and predict relapse earlier than imaging. “Created in BioRender. Lalla, M. (2026) https://BioRender.com/o9tdj76”.

From a translational perspective, MRD provides a measurable bridge between early persistence and later relapse. ctDNA profiling can detect post-treatment MRD and predict relapse earlier than imaging in some cohorts (Figure 3) [18,19]. Though ctDNA-based MRD is not synonymous with the DTP state, it is able to capture molecular heterogeneity that is associated with persister cells and offers dynamic monitoring that better accommodates the kinetics of the DTP state [54,55,56,57]. Unfortunately, ctDNA assays post-surgery and post-treatment currently lack standardization and sensitivity [54,55]. Integrating ctDNA monitoring with DTP-specific biomarkers—such as ALDH and KDM5, EMT/AXL signatures [15], and dormancy programs [7,24]—may enable more precise identification of DTPs and the design of biomarker-guided interventions.

## 7. Potential Therapeutic Strategies and Clinical Translation

Potential therapeutic strategies against persistence broadly fall into three categories: (i) preventing DTP formation, (ii) eliminating persisters by exploiting emergent vulnerabilities, and (iii) enforcing dormancy to delay or prevent outgrowth. These approaches raise distinct trial-design issues, including when to intervene (early on-treatment versus post-response), how to identify the relevant residual state (biomarkers), and which endpoints best capture residual disease biology (e.g., MRD clearance, time-to-next-treatment, metastasis-free survival).

### 7.1. Preventing DTP Formation with Rational Combinations

A central goal is to reduce the persister reservoir during the earliest treatment window, before stable resistance evolves. The chromatin-mediated drug-tolerant state described by Sharma supports combination strategies that pair tumoricidal therapy with agents that blunt epigenetic plasticity or stress-adaptation [2,25].

Combination approaches should be anchored to biomarker-defined pathway engagement because persister programs are heterogeneous and multiple tolerant states can coexist. Practical constraints include on-target toxicities of epigenetic and stress-pathway inhibitors and the need for pharmacodynamic readouts in early on-treatment samples.

### 7.2. Exploiting Vulnerabilities of Persister Cells

Persisters can acquire liabilities that are dispensable in the drug-naïve state. Therapeutic targeting of these liabilities remains challenging as substantial variability exists regarding the metabolic pathways engaged by DTPs within individual tumor types and even by distinct DTP subpopulations within the same tumor. The dynamic and reversible nature of the DTP state also necessitates precise therapeutic timing to effectively exploit these transient dependencies.

Epigenetic modulation is a core feature of drug tolerance, first described by Sharma and colleagues [2]. Consistent with this, histone demethylases such as KDM5 inhibitors and histone deacetylase (HDAC) inhibitors have demonstrated efficacy in preclinical models by impairing the DTP survival in *EGFR*-mutated NSCLC [2,26] and may be combined with targeted therapy to prevent persister cell formation.

GPX4 dependence and ferroptosis sensitivity represent a metabolic vulnerability reported across multiple models [34]. OxPhos dependence under osimertinib pressure provides another candidate liability in *EGFR*-driven models [33]. Nevertheless, these approaches carry potential risks, as normal tissues may also be susceptible to oxidative stress and ferroptosis, underscoring the need to minimize off-target toxicities [5].

Developmental and stress-response pathways that reinforce a dormancy-like state constitute another therapeutic avenue. As described previously, WNT/β-catenin signaling has been implicated in maintaining DTP survival, and preclinical studies demonstrate that dual inhibition of EGFR and β-catenin signaling prevents persister cell emergence and improves recurrence-free and overall survival [32]. However, WNT/β-catenin pathways are also imperative for normal tissue homeostasis, complicating clinical translation of this therapeutic approach.

Because vulnerabilities may be context dependent, translation benefits from stringent benchmarks: reproducibility across models, in vivo validation under clinically realistic exposure, feasible pharmacology, and a biomarker strategy to identify the liability in patient-derived material. State-matched sequential strategies—deployed after initial cytoreduction or triggered by MRD—may be more realistic than universal add-ons.

### 7.3. Targeting Microenvironmental Support and Immune Evasion

Microenvironmental growth factors and stromal interactions can protect residual NSCLC cells during therapy. Dual targeting of protective circuits such as HGF/MET and IGF signaling can reduce tolerance in experimental systems [29,37,38]. AXL is a tolerance-associated target with clinical testing in NSCLC; bemcentinib (BGB324) combined with docetaxel was feasible in a phase 1 study and motivates biomarker-driven strategies to enrich for AXL-dependent states [57].

Because quiescent and stress-adapted phenotypes can coincide with immune escape, integrating persistence-directed strategies with immunomodulation is conceptually appealing but should be evaluated carefully for timing and safety. Immune-competent models and prespecified immune endpoints can strengthen translational inference.

### 7.4. MRD-Guided Interventions and Dormancy-Directed Concepts

ctDNA-based MRD detection offers a platform for escalation or tailoring of adjuvant therapy when disease burden is lowest [18,19,20]. MRD positivity alone does not distinguish dormancy from active microscopic outgrowth; integrating ctDNA with state biomarkers remains a high-priority need.

Dormancy-directed therapy can aim to eradicate dormant cells or to enforce dormancy as a maintenance strategy. NR2F1/retinoid programs provide a mechanistic basis for dormancy enforcement. In a seminal study, ATRA induced dormancy-associated genes transiently, whereas epigenetic priming with 5-azacytidine followed by ATRA produced more durable induction of RARβ/SOX9-linked quiescence programs in an NR2F1-dependent manner [7]. More recently, 5-azacytidine plus ATRA (or AM80) stabilized dormancy programs through restored TGF-β–SMAD4 signaling and suppressed metastatic outgrowth in vivo [24].

Early clinical exploration of this approach has been reported outside of lung cancer. A pilot study in biochemically recurrent prostate cancer evaluated low-dose 5-azacytidine plus ATRA with exploratory dormancy-marker assessment [58]. Such studies are preliminary, but they provide a template for testing dormancy enforcement in low-burden disease settings with biomarker and MRD-analog endpoints.

## 8. Challenges and Future Directions

Several issues limit progress toward clinically actionable persistence/dormancy targeting. Many mechanistic studies rely on long-term treated cell lines that may not reflect in vivo pharmacology, immune pressure, or niche constraints. Persister states are transient and heterogeneous, complicating biomarker development and sampling. Finally, the pathways that correlate with therapy stress are not always the pathways that causally control survival or reactivation.

Biomarker limitations are particularly constraining. ctDNA MRD assays provide powerful clinical kinetics but depend on tumor shedding and do not specify residual state, while candidate tissue markers (e.g., AXL, ALDH, chromatin regulators) often reflect broader stress or lineage programs. Progress will likely require integrated strategies that combine MRD kinetics with tissue-based single-cell or spatial profiling, and prospective evaluation of sensitivity, specificity, and predictive value.

Near-term priorities could include: integrating MRD assays with tissue profiling in neoadjuvant/adjuvant NSCLC studies; developing in vivo models that capture dissemination niches relevant to lung cancer dormancy; and performing rigorous studies to establish causality for candidate persister regulators and reactivation pathways [5,21].

## 9. Conclusions

DTP cells and tumor dormancy provide complementary frameworks for why lung cancers recur after initially effective therapy. Evidence supports a model in which early, reversible persistence—enabled by chromatin remodeling, transcriptional plasticity, metabolic rewiring, and microenvironmental protection—creates an MRD reservoir that may remain clinically occult or eventually fuel overt relapse. Bridging mechanistic insights with clinically tractable MRD measurement, validated state biomarkers, and biomarker-guided trials designed around residual disease endpoints will be essential to translate persistence- and dormancy-directed strategies into durable patient benefit.

## Data Availability

No new data were created or analyzed in this study. Data sharing is not applicable to this article.

## Abbreviations

The following abbreviations are used in this manuscript:

NSCLC	Non-small cell lung cancer
DTP	Drug-tolerant persister
ctDNA	Circulating tumor DNA
MRD	Minimal residual disease
TKI	Tyrosine kinase inhibitors
EMT	Epithelial-mesenchymal transition
IGFR-1	Insulin-like growth factor 1
IGFR-1R	Insulin-like growth factor 1 receptor
OxPhos	Oxidative phosphorylation
ADH	Aldehyde dehydrogenase
